# Supplementary material for: Dietary Exposure to Polychlorinated Biphenyls and Dioxins and Its Relationship to Telomere Length in Subjects Older Than 55 Years from the SUN Project
Source: Nutrients. 2022 Jan 14;14(2):353. doi: 10.3390/nu14020353 (PMC8779661; doi:10.3390/nu14020353)
Supplement: Supplementary file 1 [file nutrients-14-00353-s001.zip › nutrients-1499656-supplementary.pdf]

Dietary exposure to polychlorinated biphenyls and dioxins, and its relationship with telomere length in subjects older than 55 years old from the SUN project

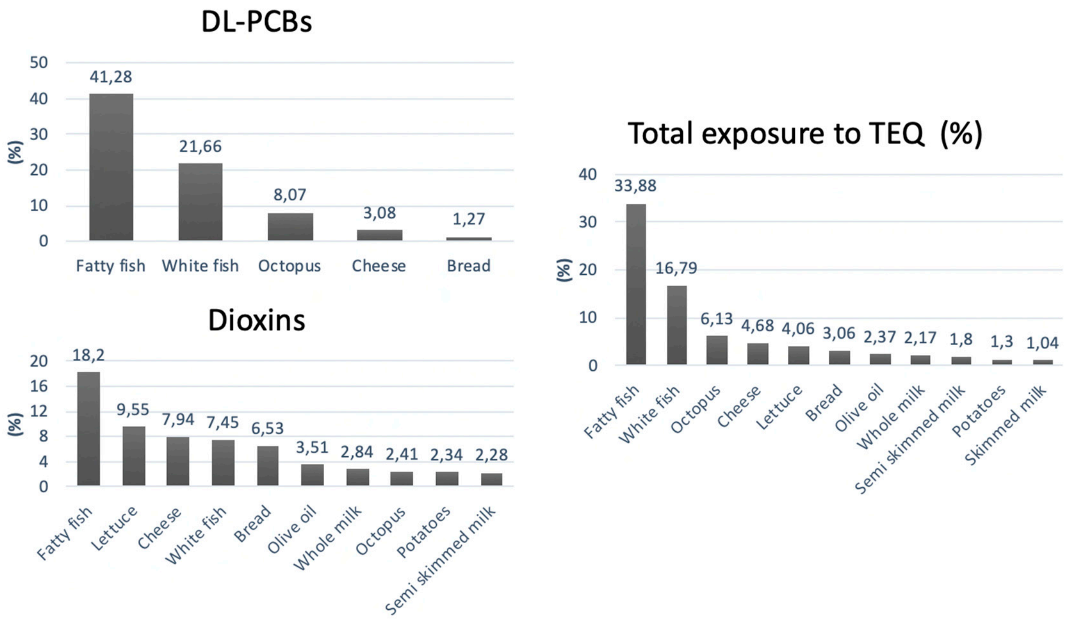

Supplementary Figure S1. Percentage of main food groups contributing to total amount of energy- adjusted DL-PCBs and dioxins consumed in 886 participants over 55 y from the SUN Cohort. Abbreviations: DL-PCBs; Dioxin-like Polychlorinated biphenyls; SUN, Seguimiento Universidad de Navarra; TEQ, Toxic Equivalents.

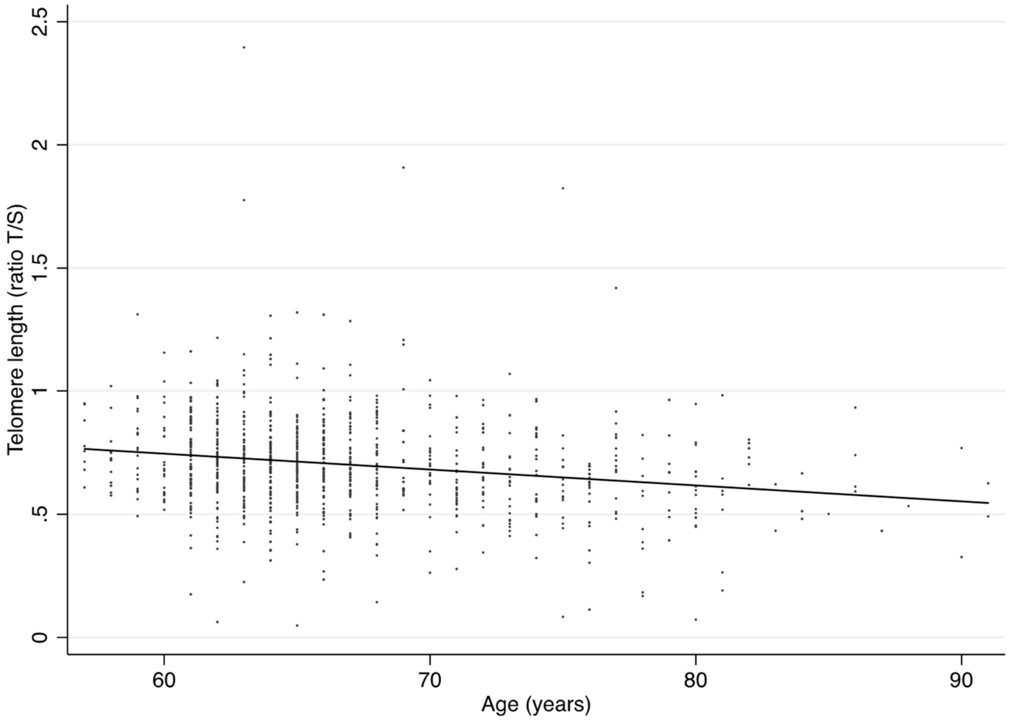

Supplementary Figure S2. Correlation of relative telomere length with age.

Effect = -0.1965, *p*-value = <0.001.

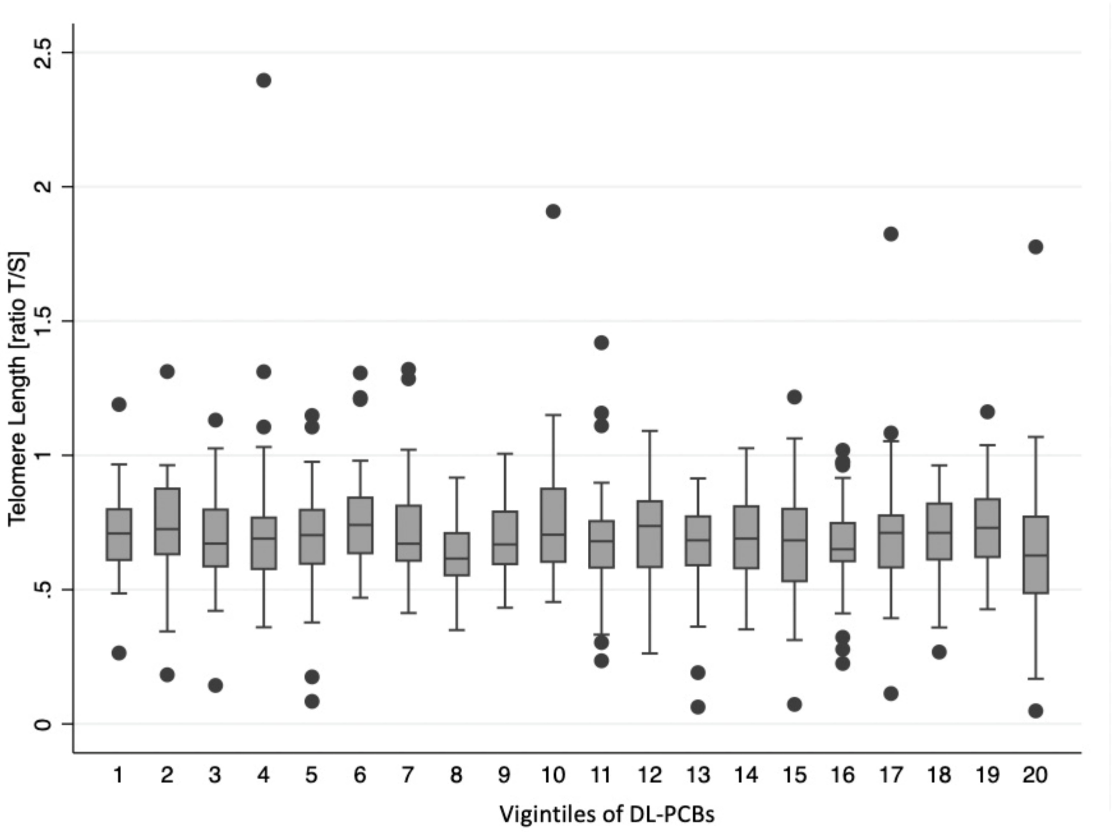

Supplementary Figure S**3**. Box plot of relative telomere length with vigintiles of DL-PCBs. Correlation coefficient inverse probability weighting-adjusted* vigintiles of DL-PCBs =-0.1896, p-value:<0.001

*Adjusted for age and sex, body mass index (kg/m^2), energy intake (kcal/d), personal history of CVD, obesity, HTA, diabetes, cancer and dyslipidemia (yes or no), educational level (year at university, continuous), smoking status (current, never, former), physical activity (MET-h/week, continuous), computer hours (continuous), TV hours (continuous), sleeping hours (continuous), sleeping/siesta (yes or no), snacking between hours (yes or no), alcohol consumption (g/d, continuous), cholesterol intake (mg/d), fiber intake (g/d, continuous), total fats intake (percentage of total energy intake of lipids, continuous), ultra-processed food consumption (servings/day, continuous), following a special diet (yes or no) and Mediterranean diet (scale 0-9, continuous).
